# Evaluating the Effects of 60°C Heating for 90 Min on Bacterial Pathogen Viability and IgG Concentration in Bovine Colostrum

**DOI:** 10.1002/vms3.70431

**Published:** 2025-06-10

**Authors:** Mostafa Moazeni, Aria Rasooli, Mohammad Nouri, Masoud Ghorbanpoor, Nader Mosavari, Amir Zakian

**Affiliations:** ^1^ Department of Clinical Sciences, Faculty of Veterinary Medicine Shahid Chamran University of Ahvaz Ahvaz Iran; ^2^ Department of Animal Health Management, School of Veterinary Medicine Shiraz University Shiraz Iran; ^3^ Department of Pathobiology, Faculty of Veterinary Medicine Shahid Chamran University of Ahvaz Ahvaz Iran; ^4^ Department of Pathobiology, Faculty of Veterinary Medicine Shahrekord University Shahrekord Iran; ^5^ Bovine Reference Laboratory, Razi Vaccine and Serum Research Institute Agricultural Research, Education and Extension Organization (AREEO) Karaj Iran; ^6^ Department of Clinical Sciences, Faculty of Veterinary Medicine Lorestan University Khorramabad Iran

**Keywords:** colostrum, heat‐treatment, IgG, *Mycobacterium avium* subsp, on‐farm batch pasteurizer, paratuberculosis

## Abstract

The purpose of this study was to investigate the effects of heat treatment on bovine colostrum at 60°C for 90 min on the viability of bacterial pathogens and IgG concentration. In the first experiment, eight batches (4.5 L) of bovine colostrum were inoculated with *Escherichia coli* (10^6^ cfu/mL), *Salmonella* Enteritidis (10^6^ cfu/mL), *Mycobacterium avium* subsp. *paratuberculosis* (MAP;10^3^ cfu/mL) and *Mycobacterium avium* subsp. *avium* (MAA;10^3^ cfu/mL). These batches were then heat‐treated using a commercial on‐farm batch pasteurization system at 60°C for 90 min. Samples were collected before inoculation, after inoculation, and at 30, 60, 75 and 90 min post‐heat treatment to assess pathogen viability. In the second experiment, batches of colostrum (10 L) were heated at 60°C for 90 min across seven replicates. Subsamples were collected before treatment and at 30, 60, 75 and 90 min during heat treatment to measure IgG concentration. The findings indicated that heating colostrum at 60°C for 30 min resulted in detectability (viability) of *E. coli* and *S*. Enteritidis in 12.5% and 25% of replicates, respectively, and after 60 min in 0% of replicates. In addition, heat treatment at 60°C resulted in the growth of MAP and MAA in 25% and 12.5% of replicates after 60 min, respectively, and 0% of replicates after 90 min. Importantly, the treatment did not significantly affect IgG concentration (5.7 mg/mL reduction after 90 min) (*p* > 0.05). According to the results, heating colostrum at 60°C for 60 min completely eliminates *E. coli and S*. Enteritidis and can render mycobacteria undetectable by culture in most situations. In situations where the control and eradication program for Johne's disease is being considered, heat treatment of colostrum at 60°C for 90 min can be beneficial without concern for a significant reduction in IgG levels.

## Graphical abstract

Heating bovine colostrum at 60°C for up to 90 min eliminates key pathogens, including ***Escherichia coli***, ***Salmonella* Enteritidis**, ***Mycobacterium avium* subsp. *paratuberculosis*** and ***Mycobacterium avium* subsp. *avium***, while preserving IgG levels. This protocol ensures microbial safety and colostrum quality, offering a practical solution for dairy herd health management.

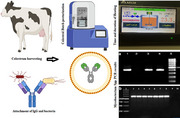

## 1 Introduction

In pregnant cows, the transfer of protective immunoglobulins from the mother to the foetus is hindered by the placental structure, which keeps maternal and foetal blood supplies separate. As a result, calves are born with very low levels of immunoglobulins and must rely on absorbing maternal immunoglobulins through colostrum (Lopez and Heinrichs 2022; Rabaza et al. 2023). Effective colostrum management is essential for the health and survival of calves, as it significantly influences their short‐ and long‐term health and performance. The importance of providing calves with an appropriate quantity of high‐quality colostrum within the first 24 h of life has been well established (Jaster 2005; Zakian et al. 2018; S. M. Godden et al. 2019; Costa et al. 2023). Feeding colostrum rich in immunoglobulins facilitates the systemic absorption of these proteins, thereby protecting the calf against systemic diseases (Weaver et al. 2000). To ensure optimal passive transfer of immunity, the accepted threshold for distinguishing high‐quality from low‐quality colostrum is 50 g of IgG per L, which corresponds to a Brix index of 22% and bacterial counts below 100,000 cfu/mL (Costa et al. 2023). One of the primary benefits of administering colostrum properly to calves is its positive impact on the productivity and longevity of cows (Gomes and Chamorro 2017). While first‐milking bovine colostrum is a vital source of immunoglobulins, nutrients, growth factors, probiotics, prebiotics and other essential immune components (Borad et al. 2021), it can also serve as an initial source that exposes calves to infectious agents such as *Mycobacterium avium* subsp. *Paratuberculosis* (MAP), *Salmonella* spp., *Mycoplasma* spp., *Listeria monocytogenes*, *Campylobacter* spp. and *Escherichia coli* (Malik et al. 2022). So, pathogenic bacteria can lead to illnesses like diarrhoea or septicaemia, and a high level of bacterial contamination in colostrum can decrease the effectiveness of Ig absorption (Sotudeh et al. 2018) through competition between microbes and IgG molecules to bind to co‐receptors on enterocytes or the physical binding of IgG and microbes in the intestinal lumen (Johnson et al. 2007). Also, many bacteria species in colostrum can negatively impact its composition, particularly the protein components, through the production of proteases (Rabaza et al. 2023).

Minimizing bacteria counts in colostrum requires an understanding of the conditions and practices that lead to their increase. Therefore, it is crucial to identify proactive measures to prevent colostrum contamination issues. In this case, comprehensive training on pre‐milking udder sanitation, milking and colostrum harvesting, correct usage and maintenance of the heat‐treating equipment and proper colostrum storage are crucial to maintain proper milking practices and improve milk quality and safety in the dairy sector (Elmoslemany et al. 2010; Martin et al. 2023). The level of colostral contamination can vary due to changes in personnel or the time available for task completion (Fecteau et al. 2002). To address this issue, heat treatment of bovine colostrum has been proposed as a method to reduce bacterial contamination and minimize the transmission of colostrum‐borne pathogens to dairy calves (S. Godden 2008; S. Godden et al. 2006). However, an experiment on pasteurizing colostrum at the typical milk pasteurization temperature of 63°C revealed significant degradation of IgG and alterations in viscosity, rendering this approach less viable (McMartin et al. 2006). The researchers in a laboratory‐based study heated 50 mL volumes of cow colostrum at temperatures ranging from 59°C to 63°C. Their findings suggested that colostrum can be safely heated at 60°C for 120 min without affecting its viscosity and IgG concentration. Johnson et al. (2007) also observed that heating colostrum at 60°C for 60 min led to improved total protein and serum IgG levels in calves.

In terms of microbial contamination of milk and colostrum, the main concern is MAP. Streeter et al. (1995) reported that as many as 22% of cows infected with MAP shed the bacteria in their milk and colostrum. Studies have indicated that pasteurizing colostrum at 60°C for 30 min can help reduce or eliminate various pathogens, with pasteurization at 60°C for 60 min being effective in eliminating MAP in most cases (S. Godden et al. 2006). S. M. Godden et al. (2015) examined the long‐term effects of heating colostrum at 60°C for 60 min on dairy farms in Minnesota and Wisconsin. They concluded that adhering to this protocol did not affect the risk of MAP transmission within the herds. The researchers identified one of the proposed possibilities as linked to the heating protocol at 60°C for 60 min, which may need revision. They suggested that one of the modifications needed in the protocol for colostrum heat treatment is to extend the heating duration, provided that field studies confirm the quality of colostrum in the new protocol.

Hence, the objective of this research was to assess the impact of heating colostrum with a commercial on‐farm batch pasteurization system at 60°C for durations of 30, 60, 75 and 90 min on IgG levels as well as the survival rates of *Escherichia coli*, *Salmonella* Enteritidis, MAP, and *Mycobacterium avium* subsp. *Avium* (MAA). Considering that infection with MAA can influence the results of the tuberculin test, and given its high genetic affinity with the agent of Johne's disease, this study also investigated the effect of this thermal protocol on the survival of this bacterium in colostrum.

## 2 Materials and Methods

The research was carried out at the PPD Tuberculin Department, Razi Vaccine and Serum Research Institute in Karaj, Iran, adhering to FDA guidelines and following the procedures described by S. Godden et al. (2006). The study took place from April 2014 to March 2016 and was approved by Shahid Chamran University of Ahvaz, Iran.

### 2.1 First Experiment

#### 2.1.1 Bacterial Spiking and Heat Treatment of Colostrum

For this experiment, 36 L of first‐milking cow colostrum were collected within 1 h after parturition. After pooling, eight homogeneous 4.5‐L batches of colostrum were prepared and stored at −20°C until the day of the experiment. Each 4.5‐L batch of frozen colostrum was then thawed using warm water (38°C ‐40°C) and transferred to a commercial on‐farm batch pasteurization system (Shirmak Co., Isfahan, Iran). To prepare standardized suspensions of local isolates of *Escherichia coli* (10^6^ cfu/mL), *Salmonella* Enteritidis (10⁶ cfu/mL), MAP (10^3^ cfu/mL) and MAA (10^3^ CFU/mL) (S. Godden et al. 2006), the protocols outlined in the Clinical and Laboratory Standards Institute (CLSI) guidelines (CLSI M07‐A10 & M24‐A2) were followed. Briefly bacterial culture is first grown in an appropriate medium under controlled conditions. The bacterial cells are then harvested by centrifugation, and the pellet is resuspended in a sterile diluent to achieve the desired concentration, typically expressed in colony‐forming units per millilitre (cfu/mL). The suspension is adjusted to the required concentration using a colony count method, and the final preparation is used for spiking. The colostrum was heated to 60°C for 90 minutes with eight replicates in this study. The colostrum reached 60°C in 20 min. Samples were collected before and immediately after bacteria were added to the colostrum, as well as at 30, 60, 75 and 90 min after reaching 60°C. At each of these specified times, samples were collected aseptically into sterile falcon tubes and then placed on ice (4°C) until the experiment was completed.

#### 2.1.2 Recovery of *E. coli* and *Salmonella* Enteritidis From Heat‐Treated Colostrum

For the selective recovery of *E. coli*, 5 mL of the sample was combined with 45 mL of EC broth (HiMedia, India). The enrichment bottles were incubated at 42.5°C for 48 h. Following incubation, 10 µL of the broth was streaked onto MacConkey agar plates (HiMedia, India), which were subsequently incubated at 37°C for 18 h (Van Kessel et al. 2004). Plates were examined for pink colonies, which indicate the presence of coliforms. Presumptive *E. coli* isolates were then confirmed through further biochemical identification tests (Markey et al. 2013).

For the selective recovery of *Salmonella* Enteritidis, the sample was first pre‐enriched in tryptic soy broth (TSB, HiMedia, India). Specifically, 25 mL of the sample was mixed with 225 mL of TSB and incubated at 37°C for 12 h. For the selective enrichment of *Salmonella*, 5 mL of the TSB mixture was transferred to 45 mL of tetrathionate broth (HiMedia, India). Following a 24‐h incubation at 42°C, 10 µL of the broth was streaked onto Xylose Lysine Deoxycholate (XLD, HiMedia, India) agar plates, which were subsequently incubated at 37°C. Plates were examined at 24 and 48 h post‐incubation for the presence of red colonies with a black centre, which are presumptive indicators of *Salmonella* spp. (Lee et al. 2015). Presumptive *Salmonella* isolates were further confirmed through additional biochemical and serological identification tests (Van Kessel et al. 2004).

#### 2.1.3 Recovery of MAP and MAA

**FIGURE 1:**
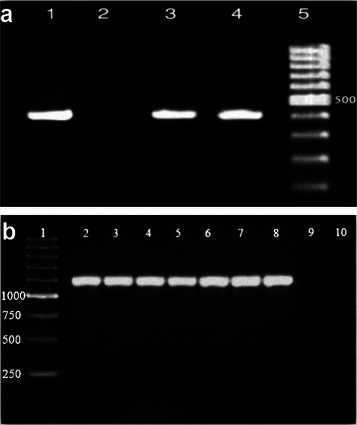
Gel electrophoresis results of PCR Assays for *Mycobacterium avium* subsp. *paratuberculosis* and *Mycobacterium avium* subsp. *avium*. **a)** *Mycobacterium avium* subsp. *paratuberculosis*: Line 1: positive control; Line 2: negative control; Lines 3 and 4: samples; Line 5: DNA ladder. **b)** *Mycobacterium avium* subsp. *avium*: Line 1: DNA ladder; Line 2: positive control; Lines 3–8: samples; Lines 9 and 10: negative controls.

The heat‐treated spiked colostrum samples from the previous steps were centrifuged at 4000 rpm for 20 min. The supernatant was then discarded, and the sediment was decontaminated with 5 mL of N‐acetyl‐L‐cysteine/sodium hydroxide (2%, consisting of 5 g/L N‐acetyl‐L‐cysteine in 3.5 M NaOH and 0.05 M sodium citrate) for 20 min, following the method of Goyal et al. (1999). After decontamination, the samples were recentrifuged at 3000 rpm for 15 min. The supernatant was again removed, and the residue was neutralized with hydrochloric acid. The residue was cultured on a slant of Herrold's egg yolk medium (HEYM) supplemented with mycobactin, as well as on HEYM without mycobactin (Thermo Fisher Scientific Inc., USA), Lowenstein–Jensen (LJ) medium supplemented with glycerol (LJG), and another slant supplemented with pyruvate (LJP) (Thermo Fisher Scientific Inc., USA). The inoculated slopes were incubated at 37°C for 16 weeks, with bacterial growth monitored at weeks 4, 8, 12 and 16. Mycobacterial growth was subsequently confirmed through acid‐fast staining and PCR (Hermans et al. 1990; Van Soolingen et al. 2001). The PCR procedure was adapted from the method described by Van Soolingen et al. (2001). In summary, bacteria from fresh subcultures on medium slants were heat‐killed and treated with acetyl trimethyl ammonium bromide, lysozyme and proteinase K, followed by chloroform extraction of DNA. The extracted DNA was then amplified with specific primers for MAP (Naser et al. 2004) and MAA (Kunze et al. 1991) according to standard procedures. For the amplification reaction MAP, primers IS900P90 (5′ GTTCGGGGCCGTCGCTTAGG 3′) and IS900P91 (5′ GAGGTCGATCGCCCACGTGA 3′) were employed to amplify a 400‐bp segment of the insertion element IS900. In addition, the MAA primers IS901 (F: 5′ GCAACGGTTGTTGCTTGAAA 3′, R: 5′ TGATACGGCCGGAATCGCGT 3′) were utilized to amplify a 1108‐bp segment (Figure 1).

### 2.2 Second Experiment

#### 2.2.1 Colostrum IgG Concentration After Heat Treatment

To investigate the effect of heat treatment on colostrum IgG concentration, we conducted heating experiments on seven replicates. In each replicate, 10 L of colostrum were poured into a pasteurization machine. The colostrum was then heat‐treated at 60°C for up to 90 min. Samples were collected before heating and at 30, 60, 75, and 90 min post‐heating. All samples were preserved at −20°C until their IgG concentration was assessed using a commercial ELISA Kit (Bio‐X Diagnostics, Belgium).

#### 2.2.2 Statistical Analysis

The results of the study were analysed using MedCalc Statistical Software (Ver.  23.0.9, Ostend, Belgium). Initially, the distribution of data was assessed using the Shapiro–Wilk test. The mean and standard deviation (SD) of IgG concentration at each time point were calculated to summarize the central tendency and variability. A non‐parametric Kruskal–Wallis Test was implied for comparing bacterial reduction across groups. A Repeated Measures ANOVA was used to evaluate changes in IgG concentration over time, given the repeated measurements within the same colostrum batches. Pairwise comparisons between time points were conducted using the Bonferroni correction to account for multiple testing. To evaluate the significance of pathogen growth across multiple time points during heat treatment at 60°C, Cochran's *Q* Test was performed. This non‐parametric test was applied to determine whether the proportion of replicates with bacterial growth significantly differed across the heat‐treatment durations (0, 30, 60, 75 and 90 min) for each pathogen (*Escherichia coli*, *Salmonella* Enteritidis, MAP and MAA). At first, the binary outcomes (1 = bacterial growth detected, 0 = no bacterial growth) were recorded for each replicate across the five time points. Then, for each pathogen, Cochran's *Q* Test was used to determine whether there was a statistically significant difference in the proportion of replicates with no bacterial growth across the time points. If Cochran's *Q* Test indicated a significant difference (*p* < 0.05), McNemar's Test with Bonferroni correction was used to perform pairwise comparisons between specific time points to identify where significant differences occurred. In addition, the proportion of replicates positive for bacterial growth was plotted against heating duration, while IgG concentration was presented on a secondary *y*‐axis. Data visualization was performed using Python (Matpotlib), ensuring clear representation of trends in bacterial reduction and IgG stability over time.

## 3 Results

### 3.1 Viability of Bacterial Pathogens

**FIGURE 2:**
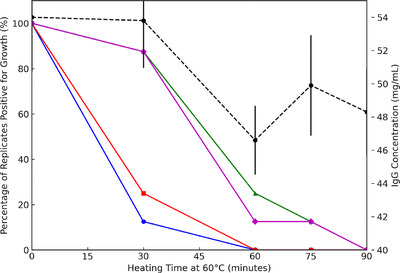
The impact of heating colostrum at 60°C over time using an on‐farm batch pasteurizer (Shirmak, Iran) on both bacterial survival (in eight replicates) and IgG concentration (in seven replicates). *X*‐axis: heating time (min) at 60°C in an on‐farm pasteurizer, ranging from 0 to 90 min. Left *Y*‐axis: The percentage of replicates positive for bacterial growth, representing pathogen survival. Right *Y*‐axis: IgG concentration (mg/mL) with error bars indicating standard deviations. Blue solid line with circles (●)—*Escherichia coli*, red solid line with squares (■)—*Salmonella* Enteritidis, green solid line with triangles (▲)—*Mycobacterium avium* subsp. *paratuberculosis* (MAP), purple solid line with diamonds (◆)—*Mycobacterium avium* subsp. *avium* (MAA) and black dashed line—IgG concentration (mg/mL).

Table 1 outlines the efficacy of heat treatment at 60°C in eliminating *Escherichia coli* and *Salmonella* Enteritidis in colostrum. All eight replicates were contaminated with the both pathogens prior to inoculation. Following 30 min of heat treatment, *E. coli* and *Salmonella* Enteritidis were undetectable in 87.5% and 75% of replicates, respectively. Importantly, no growth of either pathogen was observed in any replicate after 60 min of heat treatment, confirming that a 60‐min duration is sufficient for their complete eradication. Table 2 demonstrates the survival patterns of MAP and MAA. The findings revealed that in two out of the eight heat‐treated batches, initial contamination with MAP was present prior to inoculation. Both pathogens were detected in all replicates immediately after inoculation and remained detectable in seven replicates after 30 min of heating. By 60 min, MAP and MAA were undetectable in 75% and 87.5% of replicates, respectively. Complete elimination of both pathogens was achieved only after 90 min of heat treatment. These findings highlight the greater thermal resilience of MAP and MAA compared to *E. coli* and *Salmonella* Enteritidis, necessitating extended heating to ensure their complete inactivation. The results presented in Tables 1 and 2 are qualitative, indicating the presence or absence of bacterial growth at each time point during the heat treatment. Results of this study demonstrated a significant pathogen reduction was observed after 30 min, only 12.5% and 25% of replicates of *E. coli* and *Salmonella* Enteritidis, respectively, showed growth. (*p* < 0.05). MAP and MAA showed substantial reductions after 60 min, with 25% and 12.5% of replicates, respectively, exhibiting growth (*p* < 0.05). By 90 min, all replicates were free of bacterial growth for all pathogens. These results underscore the critical importance of a full 90‐min heat treatment protocol for the complete elimination of MAP and MAA, while shorter durations are adequate for *E. coli* and *Salmonella* Enteritidis (Figure 2).

**TABLE 1:** Viability of *E. coli* and *Salmonella* Enteritidis in eight colostrum batches (each 4.5 L) during heat‐treatment at 60°C up to 90 min in the Shirmak batch pasteurization system (Isfahan, Iran).

	Duration (min) at 60°C					
Bacterial species	Uninoculated colostrum	Inoculated colostrum	30	60	75	90
*E. coli*	C	+	+	−	−	−
	C	+	−	−	−	−
	C	+	−	−	−	−
	C	+	−	−	−	−
	C	+	−	−	−	−
	C	+	−	−	−	−
	C	+	−	−	−	−
	C	+	−	−	−	−
*Sallmonela* Enteritidis	C	+	+	−	−	−
	C	+	+	−	−	−
	C	+	−	−	−	−
	C	+	−	−	−	−
	C	+	−	−	−	−
	C	+	−	−	−	−
	C	+	−	−	−	−
	C	+	−	−	−	−

*Note*: Uninoculated colostrum: negative control; Inoculated colostrum: positive control.

Abbreviations: C = contaminated; (+) = growth detected; (‐) = growth not detected.

**TABLE 2:** Viability of *Mycobacterium avium* subsp. *avium* and *Mycobacterium avium* subsp. *paratuberculosis* in eight colostrum batches (each 4.5 L) during heat‐treatment at 60°C up to 90 min in the Shirmak batch pasteurization system (Isfahan, Iran).

	Duration (min) at 60°C					
Bacterial species	Uninoculated colostrum	Inoculated colostrum	30	60	75	90
*Mycobacterium avium* subsp. *paratuberculosis*	−	+	+	−	−	−
	C	+	+	+	+	−
	−	+	+	−	−	−
	C	+	+	−	−	−
	−	+	+	+	−	−
	−	+	−	−	−	−
	−	+	+	−	−	−
	−	+	+	−	−	−
*Mycobacterium avium* subsp. *avium*	−	+	+	−	−	−
	−	+	+	+	−	−
	−	+	+	−	−	−
	−	+	+	−	+	−
	−	+	−	−	−	−
	−	+	+	−	−	−
	−	+	+	−	−	−
	−	+	+	−	−	−

*Note*: Uninoculated colostrum: negative control; Inoculated colostrum: positive control.

Abbreviations: C = contaminated; (+) = growth detected; (‐) = growth not detected.

### 3.2 Colostrum IgG Concentration

Results indicated the baseline IgG concentration prior to heating was 54 ± 8.24 mg/mL. After 30 min of heating, the IgG concentration showed a minimal reduction to 53.8 ± 7.13 mg/mL, followed by a more pronounced decrease to 46.6 ± 5.16 mg/mL at 60 min. At 75 and 90 min, the IgG concentrations were 49.9 ± 7.55 mg/mL and 48.3 ± 6.52 mg/mL, respectively (Figure 2). Although these reductions reflect a cumulative loss of 5.7 mg/mL (approximately 10.6%) after 90 min of heat treatment, statistical analysis indicated that the decrease in IgG concentration was not significant (*p* > 0.05). This finding suggests that prolonged heating at 60°C does not compromise the immunological integrity of colostrum, preserving its key functional property for passive transfer of immunity in calves.

The Figure 2 illustrates the effect of heat treatment at 60°C on pathogen viability and IgG concentration over time. A significant reduction in the percentage of replicates positive for growth was observed for all tested pathogens as heating time increased, with complete inactivation occurring by 90 min. Meanwhile, IgG concentration remained relatively stable, with minor fluctuations but no substantial degradation. These findings suggest that heat treatment effectively reduces pathogen load while preserving IgG integrity in colostrum.

## 4 Discussion

Colostrum serves as a crucial source of nutrients, immune factors and various bioactive substances that significantly influence the health, growth and development of calves, both in the short and long term. However, it also represents the first point of contact through which neonates may be exposed to infectious agents. Asgari et al. (2022) found that only 57.3% and 66.7% of colostrum samples met the recommended thresholds for total plate count (TPC) and total coliform count (TCC), respectively. Alarmingly, just 37.3% of fresh colostrum samples satisfied all quality criteria, including IgG concentration, TPC and TCC. This indicates that many calves are at risk of receiving poor‐quality colostrum, particularly due to microbial contamination. To mitigate this risk, heat treatment has emerged as an effective method for eliminating or reducing microbial contamination in colostrum (S. M. Godden et al. 2019). Research on colostrum pasteurization has consistently demonstrated that treating colostrum at 60°C for 60 min effectively reduces or eliminates bacterial pathogens while maintaining its viscosity and IgG concentration (S. Godden 2008; Johnson et al. 2007; Donahue et al. 2012). The primary concern in colostrum pasteurization is MAP, which can lead to Johneʾs disease in ruminants (Grant et al. 2005; McDonald et al. 2005). The transmission of Johneʾs disease is well‐known to occur through the oral route, with newborn calves being the most vulnerable group. Contaminated colostrum serves as a major source of infection (Constable et al. 2016). S. Godden et al. (2006) demonstrated that heating colostrum at 60°C for 60 min led to the elimination of MAP in 75% of replicates (3 out of 4), while in one replicate, MAP was isolated for as long as 90 min. The researchers indicated that although heating at 60°C for 60 min may be adequate to eliminate MAP in most instances, these findings necessitate further investigation to confirm their reproducibility. However, in a field trial on six large commercial dairy farms, S. M. Godden et al. (2015) found that feeding calves with colostrum heated at 60°C for 60 min did not yield long‐term benefits in reducing MAP transmission risk. One explanation provided was that this time and temperature combination may not sufficiently reduce viable MAP to below an infective level in colostrum. Therefore, in the current study, by extending the routine heating time (60 min), inoculated batches of colostrum were heated at 60°C up to 90 min. The results revealed that after 30 min, *Escherichia coli* was detected in one of the eight replicates (12.5%) and *Salmonella* Enteritidis in two (25%); however, neither was present after 60 min. Accordingly, a 30‐min heat treatment at 60°C was often sufficient to eliminate *Escherichia coli* and *Salmonella* Enteritidis from bovine colostrum. There was no need for a longer heat treatment to address this bacterial contamination, as the inoculated dose in this study (10^6^ cfu/mL) far exceeded typical natural infection levels. In addition, all replicates also exhibited primary contamination. S. Godden et al. (2006) found that *M. bovis*, *L. monocytogenese*, *E. coli* and *S*. Enteritidis were undetectable at 60°C after 30 min. In the current study, treatment of colostrum at 60°C effectively eliminated MAP in six out of eight replicates after 60 min, seven out of eight replicates after 75 min and all eight replicates after 90 min. Our results are in agreement with Hesami et al. (2021), who observed significant reductions in TPC and coliform counts after heat treatment at 60°C for 90 min, with E. coli and *Salmonella* Enteritidis eliminated after 60 min. Similarly, in our study, all pathogens (*E. coli*, *Salmonella* Enteritidis, MAP and MAA) were completely eliminated after 90 min of heating, further supporting the efficacy of this protocol in pathogen reduction. Therefore, in cases where a program for controlling and eradicating Johneʾs disease in dairy herds is considered, it is recommended that, in addition to implementing other management factors, the pasteurization of colostrum consumed by calves at a temperature of 60°C for 90 min be included in the agenda.

In this study, a chemical decontamination step was implemented prior to mycobacterial isolation due to the absence of commercially available selective culture media specifically for MAP and MAA. Following the N‐acetyl‐L‐cysteine‐sodium hydroxide (NALC‐NaOH) decontamination procedure, HEYM and LJ media were employed. Previous research has indicated that certain chemical decontaminants can adversely affect mycobacterial viability unless both the decontaminant type and exposure duration are carefully regulated (Dundee et al. 2001; Bradner et al. 2013a, 2013b). According to Bradner et al. (2013b), the NALC‐NaOH decontamination method, when appropriately controlled, does not significantly compromise the viability of MAP or MAA in milk samples. Consequently, its application in the present study is unlikely to have negatively influenced the number of culture‐positive MAP or MAA samples observed across various sampling time points.

In the study, the average IgG content in colostrum was 54 mg/mL prior to heat treatment. After heat treatment at 60°C for 90 min, this level decreased to 48.3 mg/mL, representing a reduction of 5.7 mg/mL, which was considered insignificant. This finding is consistent with the results of previous studies by S. Godden et al. (2006), McMartin et al. (2006), Johnson et al. (2007), Donahue et al. (2012) and Zakian et al. (2023). However, some other researches have reported conflicting results (Elizondo‐Salazar et al. 2010; Gelsinger et al. 2014). It has been suggested that two factors including batch volume (S. M. S. M. Godden et al. 2003) and colostrum quality (McMartin et al. 2006; S. Godden et al. 2006) may influence the extent of IgG loss during heat treatment. S. M. Godden et al. (2003) observed that the loss of IgG during batch pasteurization of colostrum at 63°C for 30 min could be influenced by batch volume. Specifically, large (95 L) and medium‐sized (57 L) batches experienced 58.5% and 23.5% losses in IgG concentration, respectively. In a study by Gelsinger et al. (2014), heat treatment at 60°C for 30 min resulted in reductions of 6.5, 0.9 and 3.8 mg/mL in IgG levels for high‐, medium‐, and low‐quality colostrum, respectively. Donahue et al. (2012) conducted an on‐farm study, noting decreases of 9.8 and 6.7 mg/mL in IgG levels for raw colostrum IgG concentrations of  ≥ 80 mg/mL and 70–79 mg/mL, respectively, while only a 1 mg/mL reduction was observed for medium colostral IgG levels (50–59 mg/mL). Similarly, in the present study, heat treatment of medium‐quality colostrum (54 mg/mL) resulted in an insignificant loss of IgG. It is noteworthy that, in contrast to other research, medium‐quality colostrum was heated for 90 min in our study. Elizondo‐Salazar et al. (2010) found that heating bovine colostrum at 60°C for 30 min significantly reduced IgG_1_ levels without affecting IgG_2_. In addition, Saldana et al. (2019) reported that heating colostrum at 60°C for 30 min led to a 9% reduction in Ig concentration compared to control, with a 12% reduction when heated for 60 min.

This study provides valuable insights into the effects of heat treatment on pathogen reduction and IgG retention in bovine colostrum. While the findings are robust and provide a strong foundation for future research, some aspects warrant further exploration. First, the study utilized eight replicates for bacterial reduction and seven replicates for IgG evaluation, which aligns with similar experimental designs in this field. However, expanding the sample size in future studies could further enhance the statistical power and generalizability of the findings across diverse farm settings. Second, although colostrum collection and handling were carefully standardized, natural variability in colostrum quality (e.g., IgG concentration) is an inherent characteristic of this biological material. This variability reflects real‐world conditions and adds ecological validity to the study. Future research could build on these findings by evaluating heat‐treatment protocols across a broader range of colostrum quality metrics to further confirm their applicability under diverse conditions.

This study demonstrated that heating 4.5 L bovine colostrum at 60°C for up to 90 min using a commercial on‐farm batch pasteurization system effectively eliminates major bacterial pathogens Specifically, this heat‐treatment protocol completely eliminated *Escherichia coli* and *Salmonella* Enteritidis after 60 min and MAP and MAA after 90 min across all replicates. Importantly, the heat treatment resulted in an insignificant reduction in IgG concentration, ensuring that the immunological benefits of colostrum remain intact. These findings have significant practical implications for dairy farmers and veterinarians. Heating colostrum at 60°C for 90 min provides a simple and effective strategy to reduce the risk of pathogen transmission to newborn calves, particularly in the context of controlling diseases such as Johne's disease. The ability to pasteurize colostrum without compromising its immunological properties enhances calf health and supports herd‐level biosecurity efforts, potentially improving long‐term productivity and reducing veterinary costs. Future research should explore the broader effects of this protocol on other bioactive components of colostrum, evaluate its field‐level applicability under varied conditions, and consider its integration into routine farm management practices to optimize calf health and performance.

## Author Contributions

**Mostafa Moazeni**: methodology, software, data curation, investigation, formal analysis, writing – review and editing. **Aria Rasooli**: conceptualization, methodology, data curation, investigation, validation, formal analysis, supervision, funding acquisition, visualization, project administration, resources, writing – original draft. **Mohammad Nouri**: conceptualization, investigation, validation, supervision, funding acquisition, visualization, project administration. **Masoud Ghorbanpoor**: methodology, data curation, validation, formal analysis, writing – review and editing. **Nader Mosavari**: methodology, data curation, validation, formal analysis, writing – review and editing. **Amir Zakian**: methodology, software, writing – review and editing.

## Ethics Statement

The authors have nothing to report.

## Conflicts of Interest

The authors declare no conflicts of interest.

## Peer Review

The peer review history for this article is available at https://www.webofscience.com/api/gateway/wos/peer‐review/10.1002/vms3.70431.

## Data Availability

The data are available from the corresponding author upon request.
